# Impact of single biochar application on maize growth and water-fertilizer productivity under different irrigation regimes

**DOI:** 10.3389/fpls.2022.1006827

**Published:** 2022-11-10

**Authors:** Wei Yang, Gary Feng, Yonglin Jia, Yixuan Yang, Xiaoyu Gao, Lihua Gao, Zhongyi Qu

**Affiliations:** ^1^ College of Water Conservation and Civil Engineering, Inner Mongolia Agricultural University, Hohhot, China; ^2^ Genetics and Sustainable Agriculture Research Unit, USDA-Agricultural Research Service, Starkville, MS, United States

**Keywords:** biochar, deficit irrigation, soil water stress, maize yield, fertility index, water use efficiency

## Abstract

The improvement of soil water and nutrient availability through soil management practices are crucial in promoting crop growth and obtaining high water-fertilizer productivity under limited irrigation. In this study, a 2×4 fully randomized factorial design with two drip-irrigation regimes and four biochar rates was performed during maize crop growing seasons for a semiarid region of China in 2015 and 2016. Irrigation regimes was applied on the basis of the water lower limit of -15 kPa soil matric potential as W15 and -35 kPa as W35. Maize straw-derived biochar application rate of 0 (B0), 15 (B15), 30 (B30), and 45 (B45) t ha^-1^ was once applied to sandy loam soil in the first growing season. Our results showed that the W15 and W35 regimes generally increased soil nutrient availability and organic matter content under all biochar treatment rates for the entire growth period. In comparison, the B45-induced increase in available P and K was higher in the W15 regime than in the W35 regime during the second growing season. Furthermore, biochar treatment improved the comprehensive fertility index (CFI), leaf area index, and yield of maize. Within the same biochar treatment, the CFI value was higher in the W15 regime than in the W35 regime during the first growing season. However, the opposite was observed in the second growing season. The average irrigation water productivity (IWP) increased by 11.6%, 8.8%, and 7.8% in the W35 regime and by 15.2%, 12.9%, and 10.2% in the W15 regime for the B15, B30, and B45 treatments, respectively. Moreover, biochar treatment enhanced maize grain yield and partial fertilizer productivity (PFP) of synthetic N, P, and K fertilizers under both irrigation regimes. The highest PFP values were observed in the B15 treatment under W15. In general, a one-time application of biochar treatment at a rate of 15 t ha^-1^ in the first growing season is recommended in terms of increasing the availability of N, P, K, and organic matter in sandy loam and also improve water-fertilizer productivity under irrigation water lower limit of -15 kPa soil matric potential.

## 1 Introduction

To meet the needs of a rapidly growing global population, agricultural systems must be highly water-use efficient (Dietzel et al., 2016; Munyasya et al., 2022). Agriculture is the largest contributor to freshwater consumption, accounting for 63% of the total freshwater used in China (Du et al., 2015). In addition, the agricultural irrigation use efficiency in China is approximately 52% due to unreasonable irrigation methods, around 75% lower than that in developed countries (Kang et al., 2017). Additionally chemical fertilizer application rate in China has increased from 8.8 million tons in 1978 to 56.5 million tons in 2018, and chemical fertilizers consumed in China account for approximately 49% of the world’s total chemical fertilizer utilization. Excessive fertilization leads to low productivity since it triggers severe ecological and environmental issues such as soil pollution (Bruulsema, 2018), biodiversity loss (Li et al., 2021), and water contamination (Geza et al., 2021), threatening sustainable agricultural development (Gomiero et al., 2011). Thus, it is necessary to obtain high water-fertilizer use efficiency and protect the ecological environment through soil management practices under limited irrigation.

Biochar, a multifunctional porous material with a small particle size, high surface area, low bulk density, high adsorption capacity, and abundant carbon content, has attracted much attention because of its great potential on improving soil physicochemical properties (Khalili et al., 2020; Obia et al., 2020; Hale et al., 2021; Tian et al., 2021). Several studies have addressed the positive effects of biochar treatment on soil physicochemical properties, crop growth and yield, and water and fertilizer use efficiency (Lychuk et al., 2015; Li et al., 2018; Danso et al., 2019; Shahzad et al., 2019; Zhang et al., 2020a). In a semi-deciduous agroecological zone of eastern Ghana, Danso et al. (2019) showed that a biochar soil treatment rate of 30 t ha^-1^ is a possible viable solution for farmers to increase yield and enhance water productivity of maize under deficit irrigation. Li et al. (2018) reported that the addition of approximately 30 t ha^-1^ of biochar is a suitable rate for improving soil water-holding capacity, water and nutrient productivity, tomato yield, and cost-benefit under drip fertilization in the semiarid region of Inner Mongolia, China. Zhang et al. (2020b) reported overall improvements in soil physical quality, cucumber yield, and water fertilizer productivity in alkaline soils after biochar addition combined with daily drip fertigation in the semiarid area of Ningxia, China. Additionally, for deficit irrigation, biochar addition (20 t ha^-1^) combined with inorganic fertilizer (300 kg ha^-1^) to agricultural soils is effective in enhancing soil fertility, maize yield, water use efficiency, and economic return under low rainfall conditions in Akure, Nigeria (Faloye et al., 2019a; Faloye et al., 2019b).

For continual biochar application under limited irrigation in arid and seiarid districts, previous studies have reported improvements in crop yield, water productivity, and fertilizer use efficiency through the use of straw biochar (Faloye et al., 2019a; Danso et al., 2019; Khalili et al., 2020), cotton (Wang et al., 2020), wheat (Ramlow et al., 2019), rice (Kangoma et al., 2017), and tomato (Alkhasha et al., 2019). However, limited information is available on either medium- or long-term biochar treatment under drip irrigation fertilization with film mulch. It has been previously shown that a single application of 30 t ha^-1^ of biochar in the first year was beneficial for an increase in crop yield and soil organic matter under the reduced irrigation with mulching (Yang et al., 2020). However, the changes in topsoil nutrient availability and water-fertilizer productivity after single biochar addition under contrasting irrigation water regimes are unclear, and the fertility index based on a wide range of soil physicochemical properties is still not fully understood.

Inner Mongolia is the main region for crop production in semiarid and arid zones of China, and most soils in this region are characterized by high sand content, low organic matter content, and poor water-holding capacity (Fan et al., 2005). This has resulted in considerable leaching below the root zone. Additionally, the combination of water resource shortages and low fertilizer use has greatly restricted agricultural development in the region (Yang et al., 2017). Therefore, increasing the efficiency of current agricultural water and fertilizer resource use in cropping systems is a priority to enhance food security and improve environmental issues in regions with water scarcity.

The objectives of this study were to: (i) determine the effects of biochar application on soil nutrient availability and crop water-fertilizer productivity under different irrigation amounts, and (ii) suggest suitable irrigation water regime and biochar application scheduling to improve water-fertilizer use efficiency and better soil fertility quality for a poorer soil texture with low fertility.

## 2 Materials and methods

### 2.1 Study site

**Figure 1:**
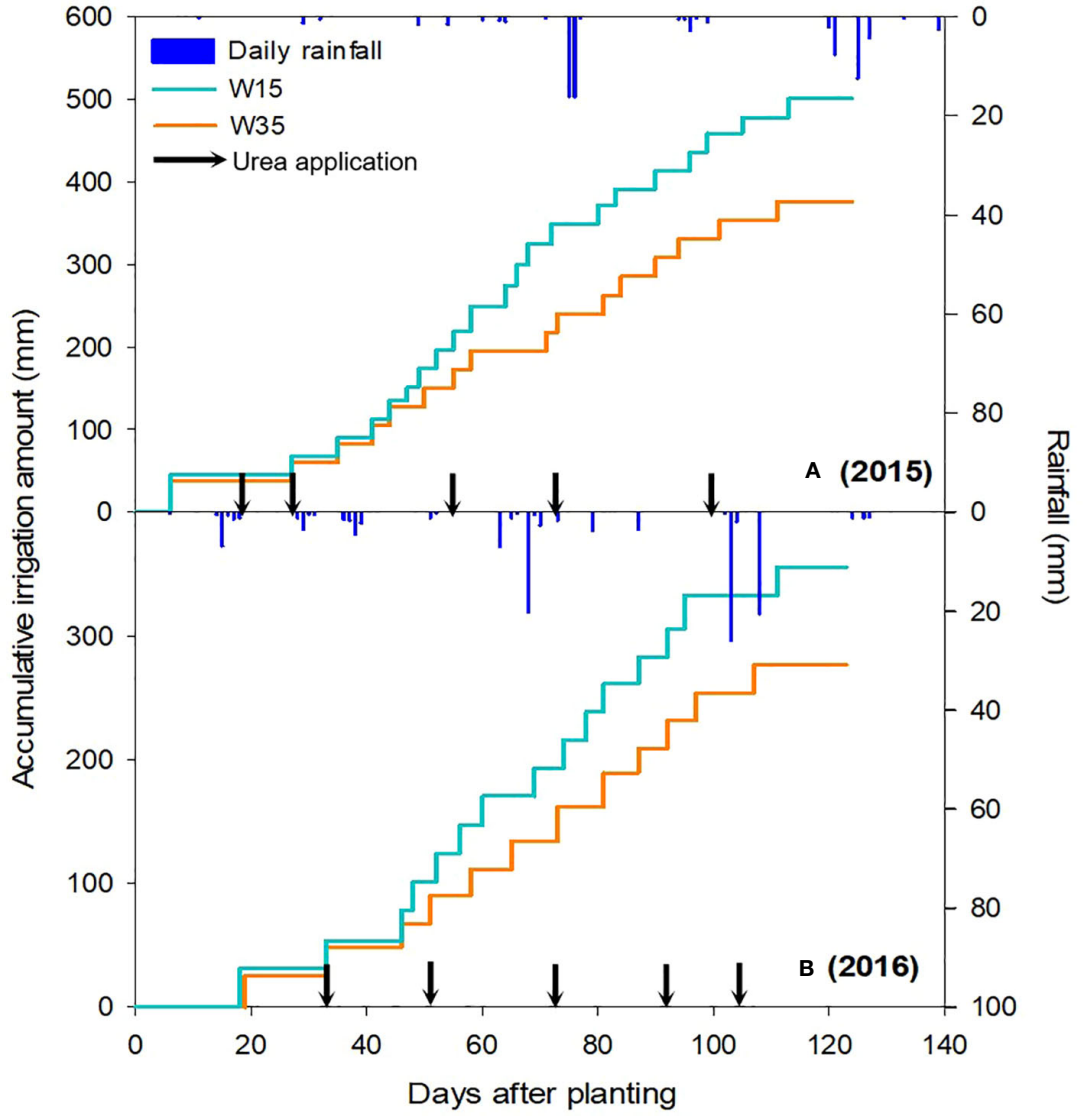
Daily rainfall and cumulative irrigation requirements at lower -15 (W15) and -35 kPa (W35) water potentials during maize growing seasons of 2015 **(A)** and 2016 **(B)**.

Field experiment was conducted during two maize cropping (2015 and 2016) seasons at the JiuZhuang Agricultural Research Experiment Station, Shuanghe County, Linhe City, Inner Mongolia, China (107°18′ E and 40°41′ N, 1042 m above mean sea level). The site belongs to a dry climate and is characterized by a few rainfall events and a high evaporation rate. According to local weather records, the highest and lowest air temperature were 40.3°C (recorded in July) and -35.3 °C (recorded in January), with an annual mean air temperature of 7.6°C. Annual frost-free and cumulative sunshine duration were approximately 130 d and 3229 h. Annual potential soil evaporation varies between 1,993 and 2,373 mm.Annual total rainfall was between 137 and 214 mm, and 75% of which (103-160 mm) was received during the crop growing season (May to September). During the maize growing season, the monthly mean air temperature was between 16.5 and 24.1°C in 2015, and 16.6 and 24.3°C in 2016, and the cumulative rainfall was 89.9 mm in 2015 and 119.1 mm in 2016 (**Figure 1**).

### 2.2 Experimental design

A 2×4 fully randomized factorial design with two irrigation regimes and four biochar rates under drip fertigation was performed during maize crop growing seasons in 2015 and 2016. Irrigation regimes were applied based on the water lower limit of -15 kPa soil matric potential as W15 and -35 kPa as W35. Biochar application rates of 0 (B0), 15 (B15), 30 (B30), and 45 (B45) t ha^-1^ was once applied to sandy loam soil for each irrigation regime in the first growing season (**Table 1**). There were 8 treatments and each treatment had 3 replicates, with a total of 24 plots. The plot dimension was 90 m^2^ (15 m×6 m) with five rows. Biochar was applied as broadcast by hands to the soil surface in mid-April 2015 and then mixed into a 20 cm soil layer with a power tiller. Biochar was not used in 2016; however, corn field operations in that year were the same as that in 2015 except for the biochar treatments. The biochar used in this study consisted of maize residue and was slowly decomposed under anaerobic conditions for 8 h at 400–500°C in a steel carbonization furnace. The principal physical and chemical properties of the biochar are listed in **Table 2**.

**Table 1:** Irrigation trigger points, biochar, and fertilizer application rate for all treatments.

Year	Factorial combinations		Fertilizer application rate (kg ha^-1^)		
	Soil matric potential (kPa)	Biochar application rate (t ha^-1^)	N	P_2_O_5_	K_2_O
2015	-15 (W15)	0	339	192	17
		15	339	192	17
		30	339	192	17
		45	339	192	17
	-35 (W35)	0	339	192	17
		15	339	192	17
		30	339	192	17
		45	339	192	17
2016	-15 (W15)	0	339	192	17
		0	339	192	17
		0	339	192	17
		0	339	192	17
	-35 (W35)	0	339	192	17
		0	339	192	17
		0	339	192	17
		0	339	192	17

**Table 2:** Physical and chemical properties for sandy loam soil and maize residue biochar.

Item	Soil	Biochar
Texture	Sandy loam	NA
Fraction of sand (%)	57.3	NA
Fraction of silt (%)	23.1	NA
Fraction of clay (%)	19.6	NA
Field capacity (cm^3^ cm^-3^)	0.25	NA
Bulk density	1.46	NA
pH	8.5	9.0
Electricity conductivity (μS cm^-1^)	318.5	NA
Organic matter (g kg^-1^)	14.5	925.7
Available N (mg kg^-1^)	65.9	159.2
Available P (mg kg^-1^)	5.3	394.2
Available K (mg kg^-1^)	184.0	783.9
Mass fraction of C (%)	NA	47.2
Mass fraction of N (%)	NA	0.7
Mass fraction of H (%)	NA	3.8
C/N	NA	67.0

NA, not available.

Maize seeds (drought tolerant cultivar ‘Ximeng No. 6’) were sown below the mulching white plastic film in early May for two growing seasons. The planting density was 56,667 plants/ha, with a row spacing of 30 cm and a plant spacing of 60 cm. The total amount of sysnthetic chemical fertilizers used for each growing season were 339 kg N ha^-1^ (synthetic compound fertilizer: 101 kg N ha^-1^; ammonium dihydrogen phosphate: 63 kg N ha^-1^; urea: 175 kg N ha^-1^), 192 kg P_2_O_5_ ha^-1^ (synthetic compound fertilizer: 17 kg P_2_O_5_ ha^-1^; ammonium dihydrogen phosphate: 175 kg P_2_O_5_ ha^-1^), and 17 kg K_2_O ha^-1^ (synthetic compound fertilizer: 17 kg K_2_O ha^-1^). Among them, 48% of total N and all P and K fertilizers were applied to the soil surface as base fertilization before sowing; the remaining chemical N as common urea (46% N)was used at the six-leaf, ten-leaf, fourteen-leaf, tassel, and fill-grain stages, with a rate of 35 kg N/ha at each growth stage (**Figure 1**).

**Figure 2:**
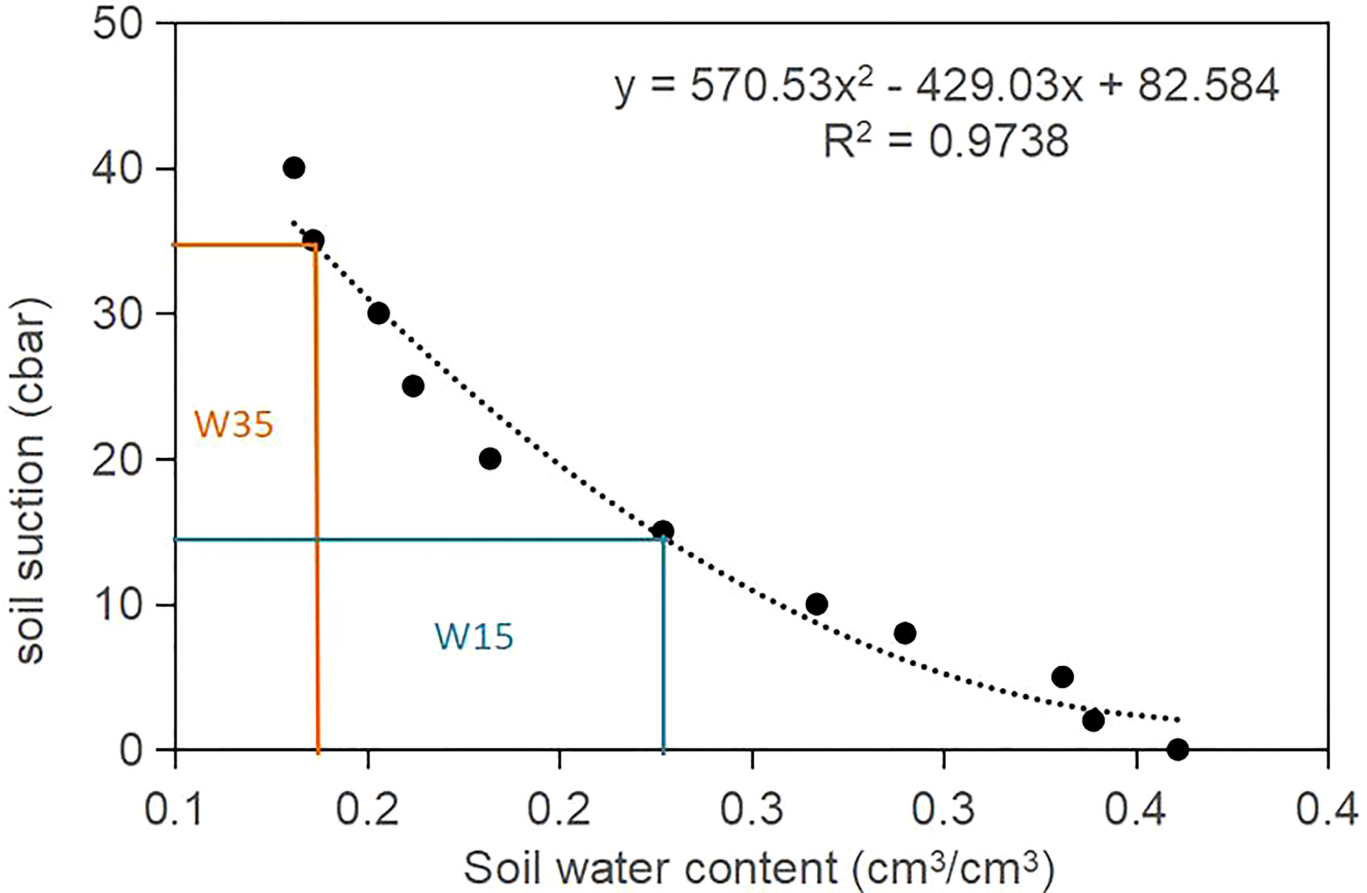
The established nonlinear relationship of sol matric potential (expressed as soil suction) and soil water content (SWC) measured at top 20 cm soil layer at the beginning of experiment in 2015.

A tensiometer (WST-2B, Beijing Autostar Technology Co., Ltd., China) was used to control the water lower limits and monitor soil matric potential under drip irrigation with plastic mulching. The tensiometer was placed at a soil depth of 20 cm near the plants under the drip irrigation line. Three tensiometers were used in the non-biochar plots for each irrigation regime. The irrigation water was used on the basis of the calculated relationship between tensiometer readings (expressed as soil water suction) and soil water content (**Figure 2**). An irrigation amount of 22.5 mm from groundwater was supplied to the plots *via* drip irrigation system when the tensiometer readings decreased to the assigned water lower limit.

The percentage of wet area was 60% and the depth of wetness was 40 cm after drip irrigation. Irrigation was triggered at approximately 10 d intervals. Irrigation was performed 17 times, with a total irrigation of 500 mm in the W15 regime and 376 mm in the W35 regime in 2015; and 13 times, with a total irrigation of 355 mm in the W15 regime and 277 mm in the W35 regime in 2016. Details of the irrigation time and irrigation amount are displayed in **Figure 1**. Drip irrigation line was placed in the middle of the two planting rows for each plastic mulching strip. The head spacing and diameter of the drip irrigation line were 300 and 16 mm, respectively, with a flow rate of 1.68 L/h for a single emitter.

### 2.3 Sampling and measurements

#### 2.3.1 Soil moisture

Soil samples at a depth of 15 cm were taken from below the drippers through soil core sampling at the three-leaf, joint, tasseling, grain-fill, and physiological maturity stages during the maize growing seasons. For each sampling, the samples were randomly selected from the middle 3^rd^ or 4^th^ rows of two film-mulched plants to avoid confounding effects. A portion of the collected samples was immediately placed in an aluminum box and then oven dried at 105°C for 24 h in the laboratory until a constant weight was reached.

#### 2.3.2 Soil nutrient and fertility index

Soil pH, nutrient availability, and organic matter from 15 cm of soil depth were measured at the physiological maturity stage of the crop. Available N was measured using a continuous flow injection analyzer (AA3, Germany); available P was extracted using 0.5 mol/L NaHCO_3_ and molybdenum antimony sulfate, and then measured using a UV-visible spectrophotometer (UV-5300PC, China); and available K was extracted *via* ammonium acetate (pH 7.0) and measured using a flame photometer (Sherwood M410, UK). Soil organic matter (SOM) was measured using potassium dichromate extraction and sulfuric acid heat oxidation (Sparks et al., 1996).

To quantitate the effect of biochar on soil fertility based on measured soil pH, nutrient availability, and organic matter, soil fertility index was presented and evaluated using a modified version of Nemero’s comprehensive pollution index classification standard as follows (Zhou et al., 2017):

(i) First, the measured values at maize physiological maturity for each treatment were normalized to eliminate the differences between dimensions according to the following equations:


$$F_{i}=M_{i}\times {\left(X_{a}\right)}^{-1}\;(M_{i}\leq X_{a})$$

$$F_{i}=1+(M_{i}-X_{a})/(X_{b}-X_{a})\;(X_{a}<M_{i}\leq X_{b})$$

$$F_{i}=2+(M_{i}-X_{b})/(X_{c}-X_{b})\;(X_{b}<M_{i}\leq X_{c})$$

$$F_{i}=3\;(M_{i}>X_{c})$$

where F_i_ is the fertility index of each individual variable: pH, available N, available P, available K, and organic matter; M_i_ is the measured value of each individual variable; X_a_, X_b_, and X_c_ are the classification indices of each individual variable (National Soil Survey Office (NSSO), 1998; **Table 3**); and i is the variable.

**Table 3:** Classification values for nutrients in soils at different classification criteria.

Classification	pH	Available N	Available P	Available K	Organic matter
criteria of variable		(mg kg^-1^)	(mg kg^-1^)	(mg kg^-1^)	(g kg^-1^)
X_a_	1.5	60	3	10	10
X_b_	6.5	120	10	100	20
X_c_	8.5	180	20	150	30

X_a_, X_b_, and X_c_ are classification index for each individual variable.

(ii) Second, Nemero’s comprehensive index classification standard was used to evaluate soil fertility quality as the comprehensive fertility index (CFI) for each treatment according to the following equation:


$$CFI=\left(\frac{N-1}{N}\right)\times \sqrt{\frac{{\left(F_{i\_min}\right)}^{2}+{\left(F_{i\_ave}\right)}^{2}}{2}}$$

where CFI is the comprehensive fertility index, F_i_min_ is the minimum fertility index among F_i_, F_i_ave_ is the average fertility index among F_i_, and N is the number of variables (where N = 5).

#### 2.3.3 Maize growth and water-fertilizer productivity

In the middle two mulching rows of each plot, the maize leaf area index (LAI) was determined at approximately 10-day intervals from sowing to harvest using the Accu PAR Model LP-80 leaf area meter (METER, Pullman, WA, USA).

At physiological maturity, aboveground plant organs (stem, leaf, cob, and grain) were harvested to determine dry weight. Grain yield was obtained from the middle two rows of each individual plot on September 23, 2015 and September 22, 2016.

Grain yield was divided by irrigation water amount to estimate irrigation water productivity (IWP) for each treatment. The partial fertilizer productivity (PFP) of N, P, and K was respectively calculated as the ratio of grain yield to amount of applied fertilizer.


$$IWP=\frac{GY}{IA}$$

$$PFP=\frac{GY}{AR}\;\;\;\;\;\;\;\;\;\;\;\;\;\;\;\;\;\;\;\;\;\;\;\;$$

where *GY* is corn grain yield (kg ha^-1^), IA is the irrigation amount (m^3^ ha^-1^), *AR* is the application rate (kg ha^-1^) for each of three chemical fertilizers during crop growing season.

### 2.4 Data processing

The mean values of the soil nutrient and crop growth variables were subjected to a two-factor analysis of variance using Statistical Product and Service Solutions (SPSS, version 19.0, IBM Corp., United States). The mean values between the treatments were compared using the least significant difference test (p< 0.05). The main and interactive effects of biochar application rate and irrigation regimes on maize yield and components were tested using ANOVA for both years 2015 and 2016.

## 3 Results

### 3.1 Soil water content dynamics

**Figure 3:**
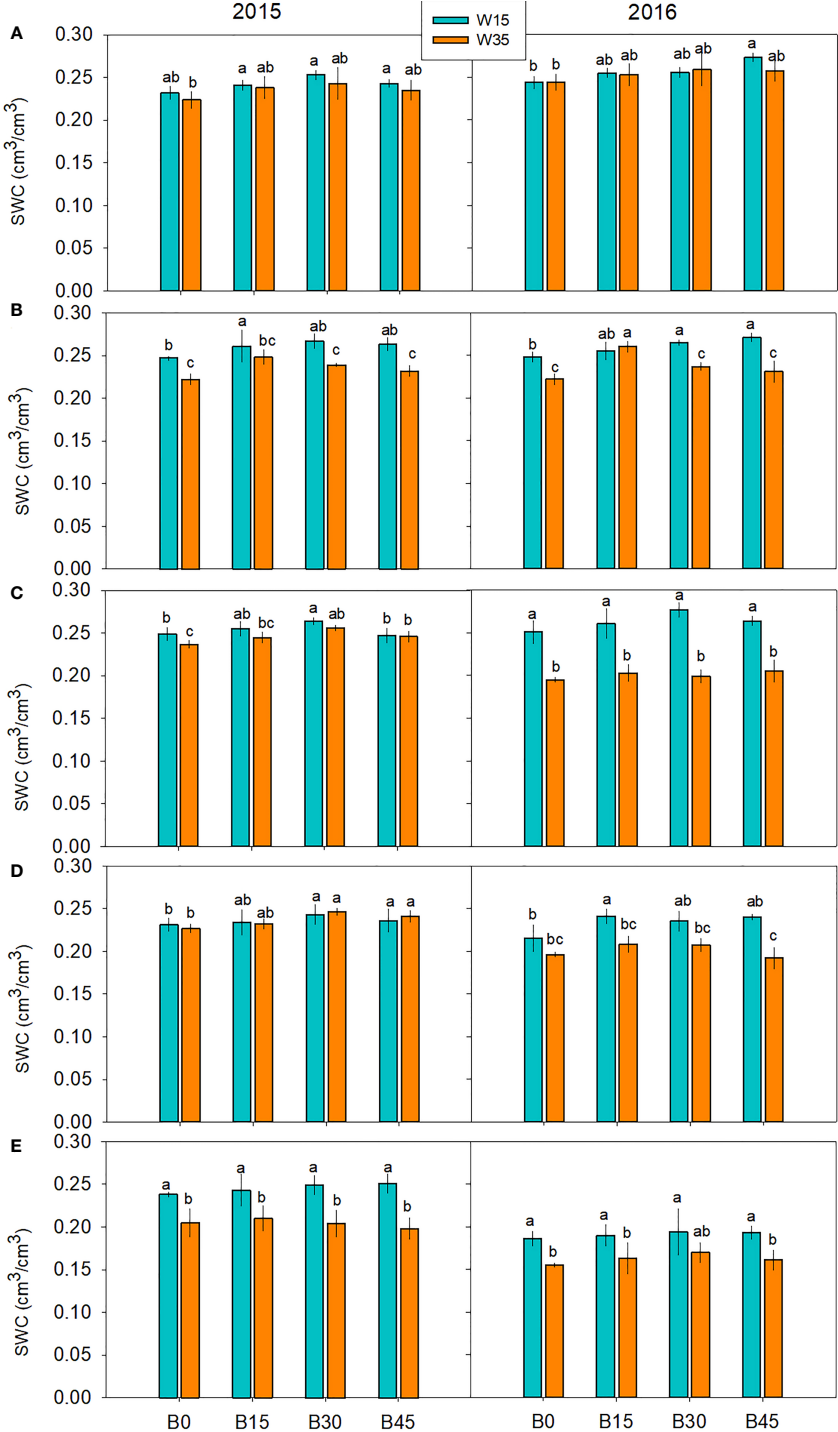
Soil water content (SWC) at top 15 cm soil layer in maize growth stages of three-leaf **(A)**, joint **(B)**, tasseling **(C)**, grain-fill **(D)**, physiological maturity **(E)** under biochar amendments (B0, B15, B30, and B45, referring as 0, 15, 30, and 45 t ha^-1^) and irrigation water lower limits at soil matric potential -15 (W15) and -35 KPa (W35) in 2015 and 2016. Different lowercase represents significant difference at P<0.05 between treatments.

For all of the maize growth periods, the SWC generally showed the increasing trend with biochar application rate under W15 and W35 in the two studied years (**Figure 3**). Compared to the non-biochar B0 plots, B30 treatment significantly led to SWC increases of 12.0%, 10.8%, and 9.1% at the three-leaf, tasseling,and physiological maturity in the first year, respectively, and 9.7%, 13.4%, and 13.6% at the three-leaf, tasseling,and physiological maturity growth periods under W35 in the second year. Within the same biochar treatments, the SWC was higher under W15 than under W35 for each growth period. It was showed that biochar could improving soil water storage for the reduced irrigation condition. The significant differences of SWCs were especially significant between W15 and W35 in physiological maturity in the first year, and tasseling, grain-fill, physiological maturity in the second year. When the four biochar treatments were averaged, the SWCs were 8.7% and 28.7% higher under W15 compared to W35 at the tasseling and physiological maturity in the first year, respectively, and were 31.3% and 17.7% higher under W15 compared to W35 at the tasseling and physiological maturity growth periods in the second year.

### 3.2 Soil available nutrient response

**Figure 4:**
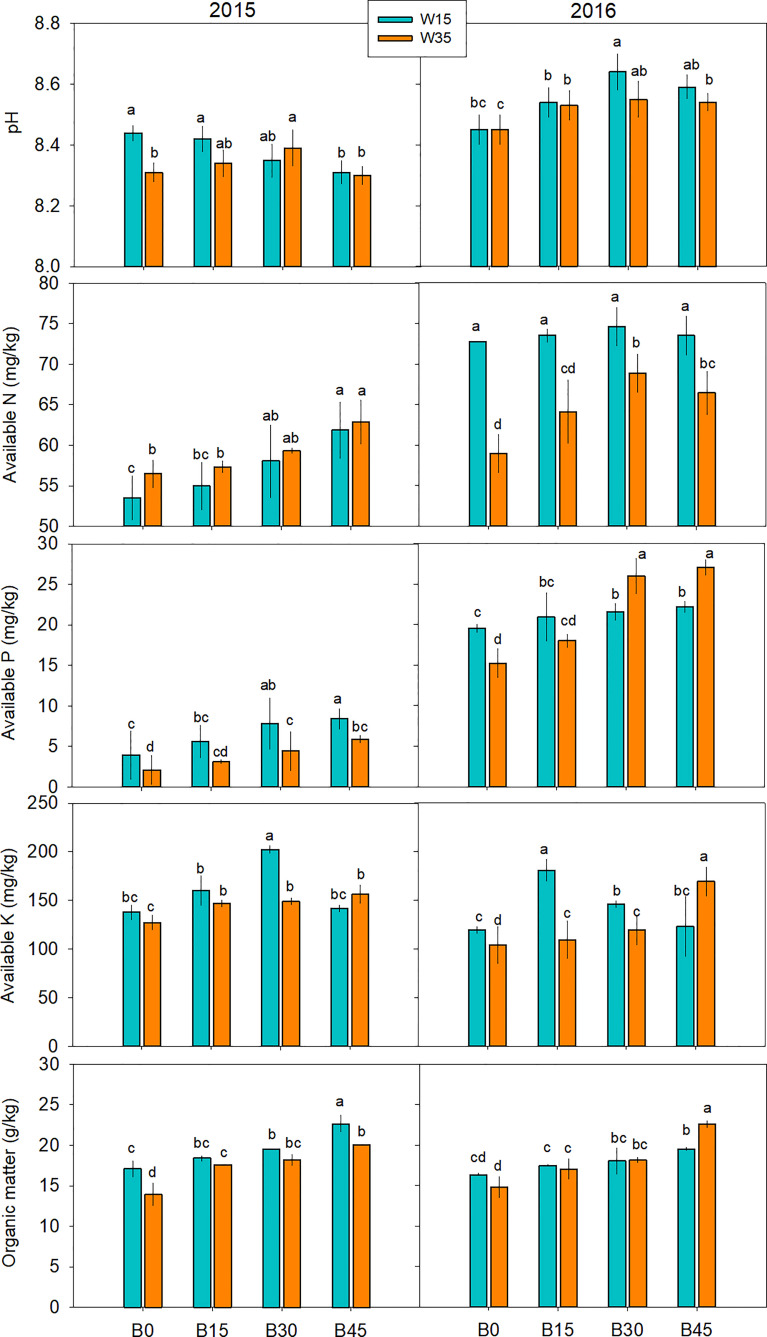
Soil pH, available nutrients, and organic matter at top 15-cm layer during maize physiological maturity stage under biochar amendments (B0, B15, B30, and B45, referring as 0, 15, 30, and 45 t ha^-1^) and irrigation water lower limits at soil matric potential -15 (W15) and -35 kPa (W35). Different lowercase represents significant difference at P<0.05 between treatments.

As shown in **Figure 4**, soil pH generally decreased in 2015 and then increased in 2016 with biochar application rate in the W15 regime, and it generally increased with biochar application rate in the W35 regime for both years. However, there were no significant differences in pH among the biochar treatments in any of the individual irrigation regimes each year. The addition of biochar resulted in an increase in available nutrients (N, P, and K) and organic matter, and the improvements in these indices were generally in accordance with the amount of biochar added. Biochar application increased the amount of available N by 2.7–12.9% under the W15 regime and 1.1–5.2% under the W35 regime in 2015, and by 1.5–9.8% under the W15 regime and 8.7–16.8% under the W35 regime in 2016. Under the same irrigation regime, the amount of available P was higher in 2016 than in 2015. Under the W15 regime, the B30 and B45 treatments significantly improved the amount of available P by 98.7% and 70.9% in 2015, and 10.5% and 13.6% in 2016, respectively. Under the W35 regime, the B30 and B45 treatments significantly improved the amount of available P by 111.9% and 133.3% in 2015, and 70.8% and 77.7% in 2016, respectively. The maximum levels of available K were obtained in the B30 and B15 treatments under the W15 regime in 2015 and 2016, respectively. There were significant differences in the amount of available P among B15, B30, and B45 treatments under the W35 regime in 2015, the values of which were significantly higher than those of the B0 treatment. When biochar was added, a significant enhancement of organic matter was reported, ranging from 7.7–24.6% under W15 and 7.1–19.3% under W35 in 2015; and 17.6–30.4% under W15 and 14.9–52.3% under W35 in 2016.

### 3.3 Soil fertility index difference

**Figure 5:**
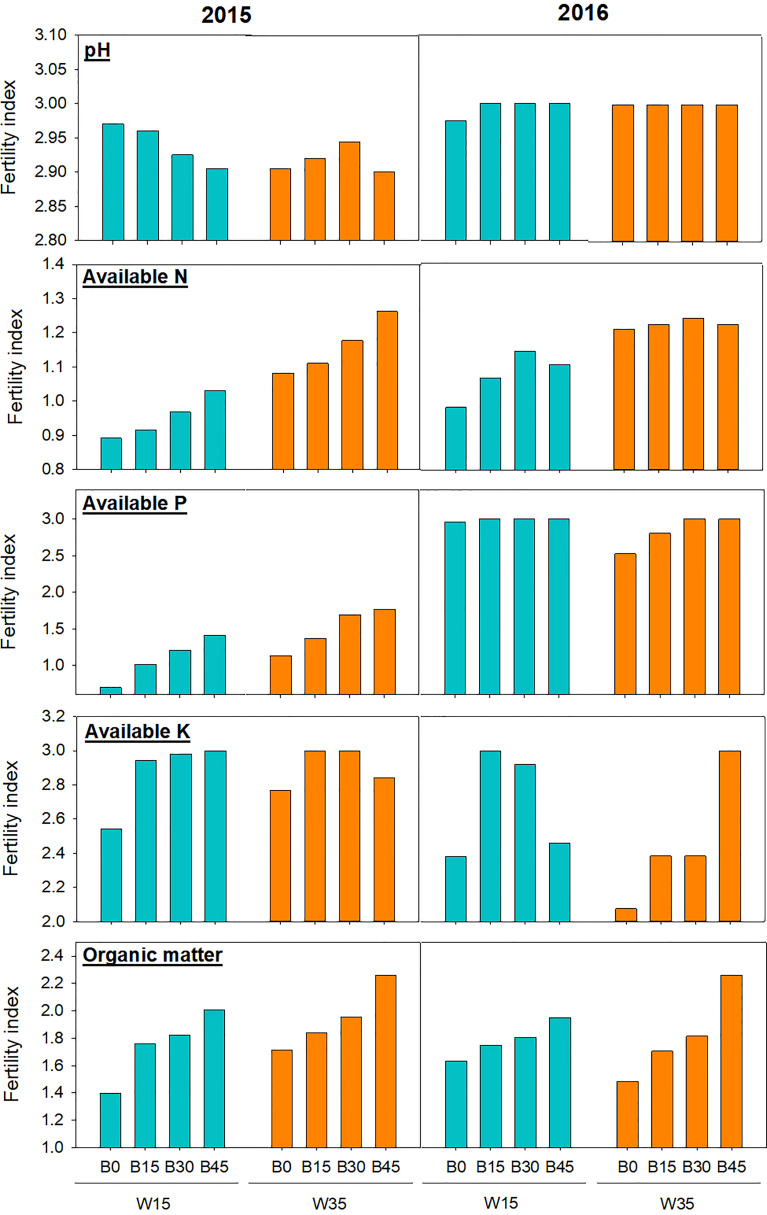
Soil fertility index for the individual variable (pH, available nitrogen (AN), available phosphorus (AP), available potassium (AK), and organic matter (OM) in the top 15-cm soil layer during maize physiological maturity stage at biochar application rates (0 (B0), 15 (B15), 30 (B30), and 45 (B45) t ha^-1^) applied in irrigation water lower limits at soil matric potential -15 (W15) and -35 kPa (W35) each year. Different lowercase represents significant difference at P<0.05 between treatments.

As shown in **Figure 5**, the fertility index for each soil variable was generally improved by biochar application in the W15 and W35 irrigation regimes. The higher the biochar application rate, the higher the fertility index for these variables. In 2015, the fertility index for available N was increased from 0.94 to 1.03 under the W15 regime, and 0.89 to 1.03 under the W35 regime. The fertility index for available P was also increased by biochar treatment under the W15 and W35 regimes; this value was greater under the W15 regime than under the W35 regime in the first year. In 2016, the fertility index for available P in the B15, B30, and B45 treatments increased under the W35 regime; the greatest fertility index was obtained at the W30 regime. The fertility index for available K increased under the B15 and B30 treatments. Under the same treatments, the fertility index for organic matter was lower under the W15 regime than under the W35 regime in the first year; however, the opposite results were observed in the second year.

**Figure 6:**
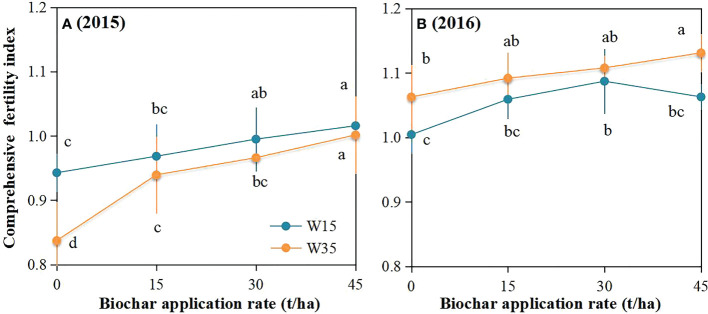
Comprehensive fertility index (CFI) for these five variables in the top 15-cm soil layer during maize physiological maturity stage at biochar application rates (0 (B0), 15 (B15), 30 (B30), and 45 (B45) t ha^-1^) applied in irrigation water lower limits at soil matric potential -15 (W15) and -35 kPa (W35) for 2015 **(A)** and 2016 **(B)**. Different lowercase represents significant difference at P<0.05 between treatments.

Comprehensive fertility index (CFI) generally increased with biochar application rate under the W15 and W35 regimes (**Figure 6**). Under the same irrigation regimes, CFI in the second growing season was higher than that in the first growth season at the same biochar rate. Under the W35 regime, biochar rates of 30 and 45 t ha-1 significantly improved the CFI by 19.6% and 6.6% in the first year, and 6.4% and 3.6% in the second year, respectively, compared to the non-biochar plots. Without biochar application, the CFI was only 11.9% and 6.6% higher under the W15 regime than under the W35 regime in 2015 and 2016, respectively. With a biochar rate of 45 t ha-1, the CFI was 5.3% lower under the W15 regime than under the W35 regime. However, there was no significant difference between the W15 and W35 regimes for biochar rates of 15 and 30 t ha-1 in each growing season.

### 3.4 Maize growth and yield traits

**Figure 7:**
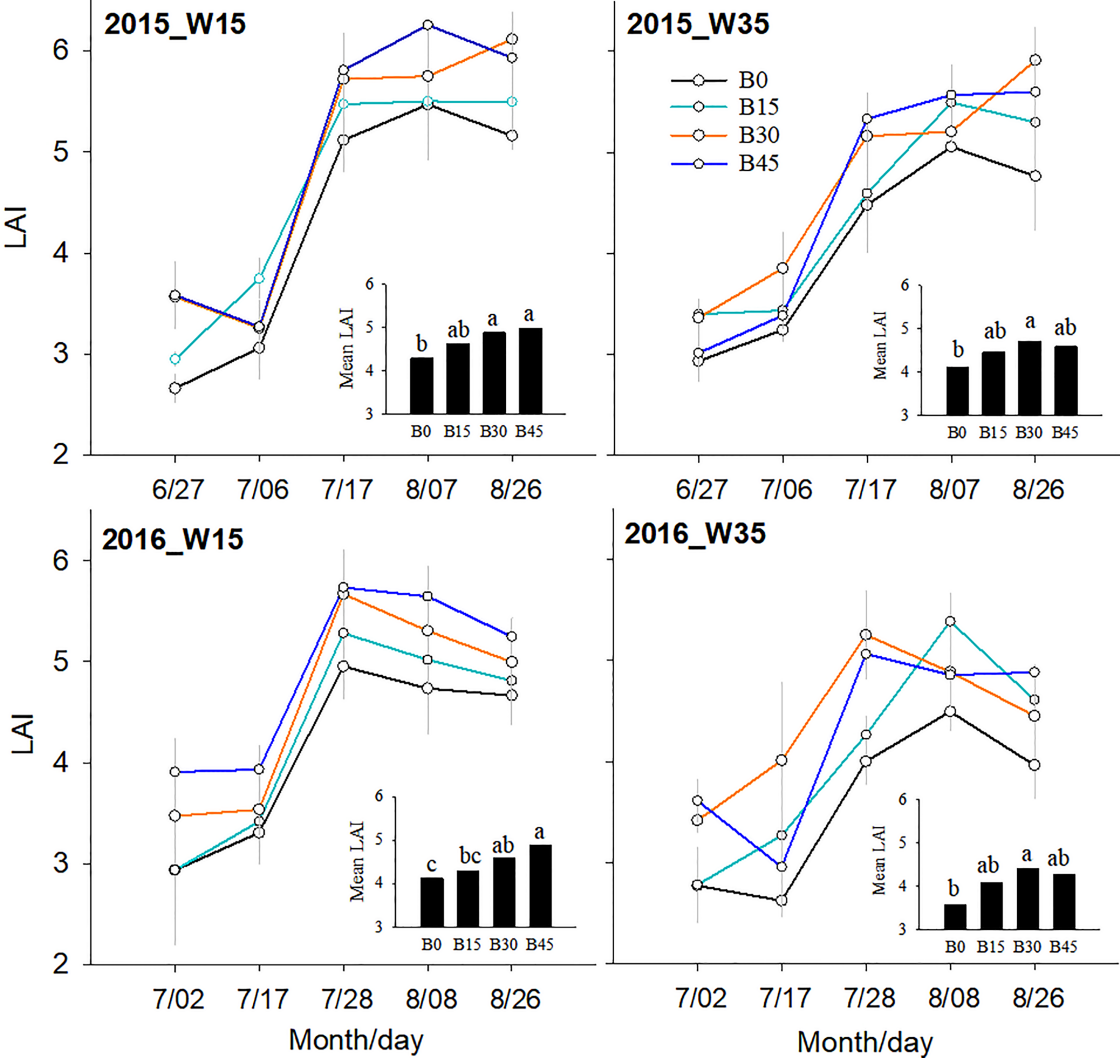
Leaf area index (LAI) for biochar amendments (B0, B15, B30, and B45, referring as 0, 15, 30, and 45 t ha^-1^) and irrigation water lower limits at soil matric potential -15 (W15) and -35 kPa (W35). Different lowercase represents significant difference at P<0.05 between treatments.

For each of the five samplings in both years, LAI values under the W15 regime were generally greater than those under the W35 regime within the same treatment (**Figure 7**). The LAI increased by biochar treatments ranged from 12.0% to 33.1% for the four sampling dates under the W15 regime. Relative to the B0 plots, the B15, B30, and B45 treatments increased LAI by 8.5%, 14.7%, and 11.8% under the W35 regime, respectively, and 7.9%, 13.7%, and 15.8% under the W15 regime in the first year, respectively, when averaged across the four measurements. Similarly, LAI values increased by 13.8%, 23.3%, and 19.7% under the W35 regime and by 4.2%, 11.6%, and 18.8% under the W15 regime, respectively, in the second year.

Maize growth indices and harvest index generally increased with increasing rates of biochar application in the two irrigation regimes. Grain yield components and aboveground biomass were greater under W15 than under W35 within the same biochar treatments for both years (**Table 4**). Compared to the B0 plots, the B15, B30, and B45 treatments led to a yield increase of 16–21% when averaging the W15 and W35 regimes in 2015. In the first year, compared to the B0 plots, the grain number in the B15, B30, and B45 plots increased by 15.4%, 14.8%, and 22.0% under W35, respectively, and 10.8%, 13.4%, and 7.7% under W15. Similarly, these improvements in grain number were found in plots treated with biochar under W15 and W35 in the second year. The grain weight did not differ significantly between biochar treatments in the W15 and W35 regimes in both years, indicating that the yield increases of the biochar amendment were principally attributed to an increase in grain number while maintaining a higher grain weight for those two irrigation regimes.

**Table 4:** Maize agronomic traits and yield components under biochar amendments (B0, B15, B30, and B45, referring as 0, 15, 30, and 45 t ha^-1^) and irrigation water lower limits at soil matric potential -15 (W15) and -35 kPa (W35).

Year	Irrigation regime	Biocharamendment	Aboveground biomass (t ha^-1^)	Grain No. per cob	100-grain weight (g)	Grain yield (t ha^-1^)
2015	W15	B0	36.7 ± 1.1 b	656 ± 13 d	40.92 ± 2.31 ab	12.80 ± 1.10 b
		B15	39.0 ± 0.6 a	727 ± 10 bc	41.63 ± 2.80 a	14.93 ± 2.12 a
		B30	36.7 ± 0.7 b	744 ± 16 b	40.97 ± 1.52ab	14.53 ± 1.80 a
		B45	39.3 ± 1.2 a	707 ± 22 cd	42.87 ± 1.73 a	14.47 ± 1.50 a
	W35	B0	32.3 ± 1.3 d	646 ± 24 d	38.98 ± 2.11 b	12.01 ± 0.32 b
		B15	36.8 ± 2.1 b	746 ± 22 b	41.11 ± 1.90 a	14.58 ± 1.43 a
		B30	35.6 ± 1.7 bc	742 ± 31 b	40.48 ± 0.65 ab	14.28 ± 1.17 a
		B45	34.9 ± 1.4 cd	788 ± 18 a	37.68 ± 1.12 b	14.05 ± 0.79 a
**ANOVA**						
Irrigation regime			*	ns	*	ns
Biochar amendment			ns	*	ns	*
Irrigation regime × biochar amendment			ns	ns	ns	ns
2016	W15	B0	31.2 ± 0.4 b	720 ± 18 d	34.77 ± 1.2 a	11.90 ± 1.41 a
		B15	33.4 ± 1.1 a	782 ± 12 a	34.73 ± 2.81 a	12.97 ± 1.23 a
		B30	31.1 ± 0.7 b	749 ± 9 bc	35.83 ± 1.32 a	12.71 ± 1.07 a
		B45	33.7 ± 0.9 a	740 ± 23 c	35.22 ± 0.56 a	12.31 ± 1.23 a
	W35	B0	30.1 ± 1.2 b	736 ± 17 c	33.92 ± 1.72 b	11.88 ± 0.87 a
		B15	31.2 ± 2.3 b	746 ± 20 bc	35.89 ± 0.92 a	12.67 ± 1.21 a
		B30	30.2 ± 1.9 b	767 ± 15 ab	33.89 ± 1.21 b	12.37 ± 0.57 a
		B45	30.1 ± 0.8 b	753 ± 14 bc	34.12 ± 0.74 ab	12.18 ± 0.89 a
**ANOVA**						
Irrigation regime			ns	**	**	ns
Biochar amendment			ns	*	ns	ns
Irrigation regime × biochar amendment			ns	ns	ns	ns

Different lowercase represents significant difference at P<0.05 between treatments. ns, non-significant. ^*^P<0.05. ^**^P<0.01. Biochar was only added in 2015.

### 3.5 Water-fertilizer productivity

**Figure 8:**
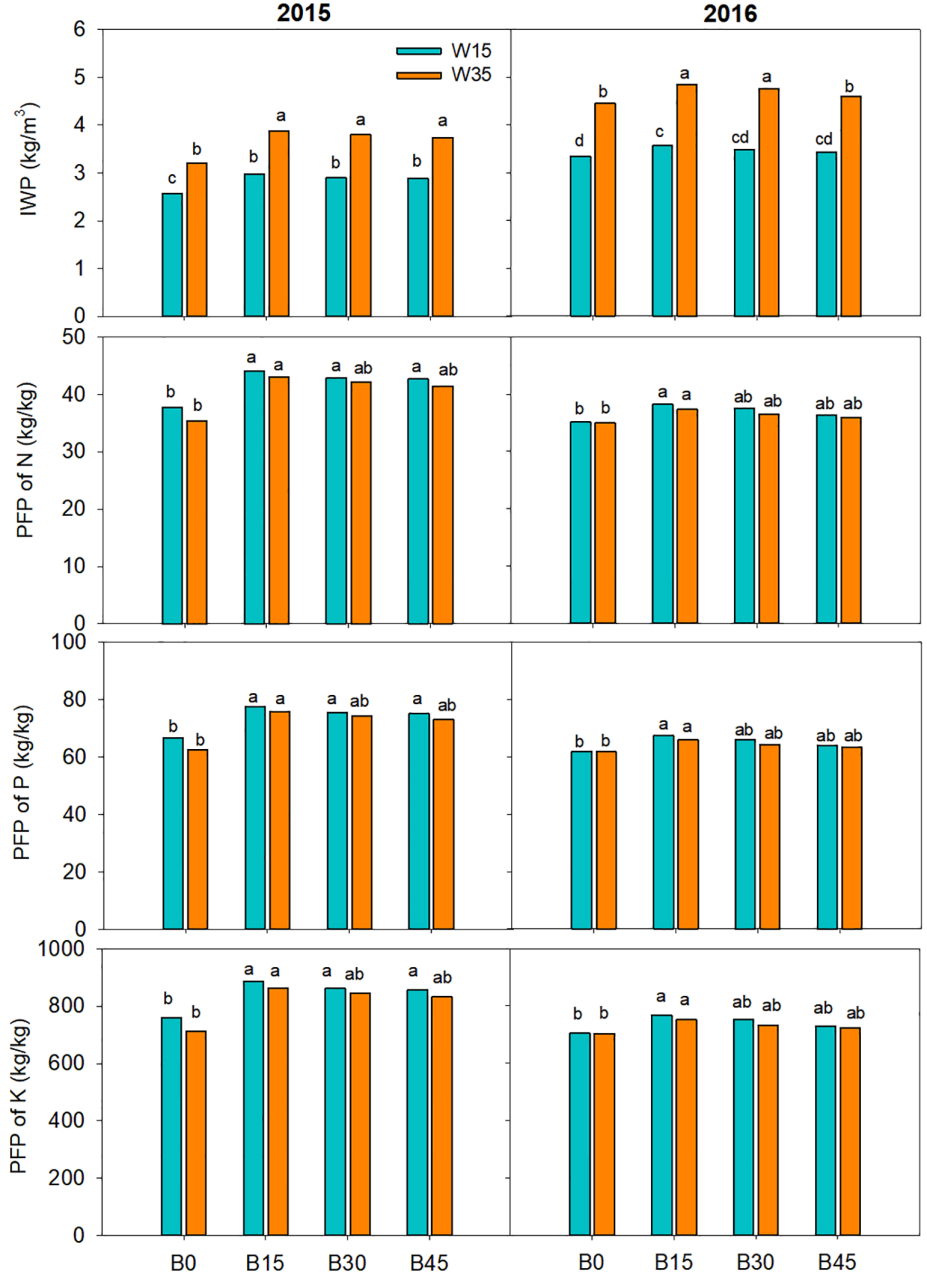
Irrigation water productivity (IWP) and partial fertilizer productivity (PFP) of nitrogen, phosphorus, potassium for maize growth under biochar amendments (B0, B15, B30, and B45, referring as 0, 15, 30, and 45 t ha^-1^) and irrigation water lower limits at soil matric potential -15 (W15)and -35 kPa (W35) in 2015 and 2016. Different lowercase represents significant difference at P<0.05 between treatments.

There was greater irrigation water productivity (IWP) in the biochar treatments than in the non-biochar control plots under the W15 and W35 regimes, as shown in **Figure 8**. In the first year, the B15, B30, and B45 treatments led to IWP increases of 21.4%, 18.9%, and 17.0% under the W35 regime, respectively; and 16.6%, 13.5%, and 13.0% under the W15 regime, compared to the B0 treatment. The IWP values in the W35 regime were significantly superior to those in the W15 regime within the same biochar treatments. For B0, B15, B30, and B45 treatments, the IWP increased by 23.1%, 28.2%, 29.0%, and 27.4% in the W35 than those in the W15 in the second year, respectively, and by 31.0%, 28.2%, 27.7%, and 29.9% in the W35 than those in the W15 in the second year.

The same trend was observed between amendment on partial fertilizer productivity (PFP) of N, P, and K in either the W15 or W35 regimes for both years. In the first year, the B15, B30, and B45 plots significantly increased PFP by 21.3%, 18.9%, and 17.0% under the W35 regime, respectively; and 16.6%, 13.5%, and 13.1% under the W15 regime, respectively, compared to the B0 plots. However, the PFP of N, P, and K among two irrigation regimes did not differ significantly within same biochar treatment for these studied years.

## 4 Discussion

### 4.1 Biochar effects on soil water content under different irrigation amounts

Several reviews and meta-analyses have addressed the effects of biochar on soil physicochemical properties, nutrients, and water fertilizer productivity under different irrigation regimes (Blanco-Canqui, 2017; Zheng et al., 2017; Gao et al., 2019; Razzaghi et al., 2020; Guo et al., 2021). This study supported some of the previous findings and demonstrated some novel insights on the effects of a one-time biochar application on soil nutrients, crop growth, and water-fertilizer use efficiency under the limited irrigation. Over a two-year field experiment, it was observed that a higher irrigation amount under the W15 regime and biochar addition improved SWC up to a depth of 15 cm (**Figure 3**). Biochar did not significantly increase SWC in the W35 regime within the two growth periods. With the increase of biochar application rate in W35, the SWC of each treatment gradually decreased, indicating that the role of biochar in improving SWC was more effective in higher irrigation amounts. W35 was the treatment with few irrigation water, and it was not irrigated in mazie maturity, the soil was mainly characterized by the extremely dehydrated. Biochar application increased soil porosity under severe drought, the more dose in biochar, the greater in soil porosity within a certain range. When soil pore arrived to the extreme shortage of soil water, the percentage volume occupied by the soil air increased, enhancing the soil evaporation intensity, and eventually led to the gradual decrease of SWC.This increase in SWC can be partly explained by biochar-induced soil pore structure (Liu et al., 2021). Biochar is a porous and carbon-rich material, and its application improves soil hydrological properties (Razzaghi et al., 2020). The sandy loam used in the experiment is characterized to have poor water-holding capacity and higher water infiltration rate (**Table 2**). Biochar has small particle sizes, can enter larger pores, and alter soil water (Yan et al., 2019). Thus, its application to topsoil can encourage the downward flow of water and increase water-holding capacity (He et al., 2020; Obia et al., 2020). Additionally, biochar contains higher salinity, its addition increases soil salinity. The increase of soil salinity improve the ability of soil to absorpt moisture, which is conducive to slowing down the evaporation of soil water, so as to improve soil moisture content.

### 4.2 Interaction effect of irrigation regimes and biochar amounts on soil nutrients

In addition to SWC, soil pH, and nutrient availability are critical for soil quality and crop production under different irrigation regimes. Under the W15 and W35 irrigation regimes, there were no significant differences in soil pH between the biochar treatments in the first year (**Figure 4**). In the second year after biochar application, pH increased with increasing rates of biochar amendment, which is consistent to measurements by Lychuk et al. (2015), who observed that biochar additions of 8 and 20 t ha^-1^ improved the pH of the upper 20 cm of Amazonian oxisol. Streubel et al. (2011) reported that soil pH increased 0.4 to 0.8 units for soils amended with 39 kg ha^-1^ of the herbaceous biochars. The soil in the study is alkaline soil; the alkaline nature of the straw biochar exchanges H^+^ with the surrounding soil, causing an increase in soil pH.

Biochar amendment performed only in the first growing season did not significantly affect available N in either the W15 or W35 regimes (**Figure 4**). However, the available N in biochar-amended soils was generally greater than that in the non-biochar control, especially at the three-leaf stage in the second year. In comparison, the available N in the W35 regime was greater than that in the W15 regime within the same biochar amendment. This indicates that biochar addition in the first year still had a positive effect on improving soil N availability under limited irrigation conditions. Possible reason for this is that the greater SWC in the W35 regime is beneficial to soil organic N decomposition during the early growing season (Abbas et al., 2020). Another explanation was that the negative charge and complex pore structure carried by the rich oxygen-containing functional groups on the surface of biochar, which endows the biochar with large cation exchange capacity and strong adsorption capacity, improving the potential of soil to absorb the available N as NH_4_-N or NO_3_-N (Faloye et al., 2019a). Meanwhile, applying biochar to the soil can slowly release soil nutrients, reduce the leaching loss and fixed loss of soil nutrients, and thus improve the content of soil available nutrients under limited irrigation.

Available P and K increased with increasing biochar application rate for all sampling dates under the W15 and W35 irrigation regimes (**Figure 4**). In especial, the enhancement of P availability was critical in improving soil nutrient quality when biochar was amended under different irrigation conditions. It further showed that higher dose of biochar application with high available P content was to the benefit of activation of P in a sandy loam soil with lower available P in the study. In contrast, available P was greater under the W15 regime than under the W35 regime at the same biochar amendment; this difference was especially obvious in the second growing season. The could be mainly related to the cycle of freezing and thawing in the studied region (Sui et al., 2022). Biochar application in the first growing season could hold more soil P in soil by enhancing the stability of soil aggregates and reducing the release of organic matter, so as to reduce the P loss as leaching and runoff during the melting period. Additionally, Biochar can alter the activity of soil microorganisms involved in organic phosphorus hydrolysis and inorganic phosphorus dissolution by releasing phosphatases and low molecular organic acids, thereby increasing phosphorus bioavailability. Our results are partly supported by those of previous reports (Zhang et al., 2013; Blanco-Canqui, 2017). Biochar increased the available K under two irrigation regimes. This is mainly related to the adsorption of K+ by electrostatic attraction on biochar-soil surface (Abbas et al., 2020). In addition, the effect of biochar on soil pH will in turn change the activity of soil microorganisms, and then stimulate the dissolution of soil potassium-containing minerals, thus increasing the content of available K (water-soluble and exchangeable K) in soil (Kangoma et al., 2017).

The calculated CFI based on these five soil parameters also improved after the addition of biochar (**Figure 6**). The CFI was generally increased with increasing biochar application rate for each irrigation regime, the trend was accorded with results of Yang et al. (2022). Within the same biochar treatment, the CFI value was higher in the W15 regime than in the W35 regime in the first year, whereas these CFI values between W15 and W35 were not significantly, which indicated irrigation regime did not significantly change CFI based measured these five variables in the study. However, in the second year after biochar addition, the CFI value was lower in the W15 regime than that in the W35 regime for same biochar application; the difference was especially significant for soil amended with 45 t ha^-1^. Biochar has much more minerals, and it can increase soil nutrients and organic matter when it was applied to sandy loam soil under reduced irrigation in semiarid region (Danso et al., 2019). However, biochar application may lead to fixation of soil inorganic nitrogen and mineralization of organic carbon as CO_2_ release due to better soil moisture under well-watering circumstance (Zheng et al., 2017; Zhang et al., 2020a), reducing content of inorganic nitrogen and increasing loss of organic carbon in soil, thus restraining the availability of soil available nutrients and their CFI values.

### 4.3 Biochar effects on topsoil organic matter under varied irrigation amounts

For each sampling over the two-year study, biochar amendments of B15, B30, and B45 generally increased SOM content at the top 15cm soil by 14–52% for the W15 and W35 regimes. The increasing trend of SOM content was in line with increasing biochar application rate (**Figure 4**). This reported range was partly in agreement with Liu et al. (2016), who found that a biochar application rate of 20–60 t ha^-1^ led to an SOM increase of 23–59%. Dong et al. (2019) and Yang et al. (2020) also reported an increase of 18–62% and 12–16%, respectively, after the addition of biochar. The reported difference in SOM increase through biochar application is possibly related to cropping systems, soil texture, field management, and climate conditions. In this study, the increase in SOM was mainly attributed to the high organic carbon content of poorly pyrolyzed biochar (**Table 2**). Moreover, biochar has positive priming effects on soil microbial activity and root growth (Danso et al., 2019; Zahra et al., 2021), both of which may regulate SOM turnover, since more microbial activity could accelerate SOM mineralization. The SOM under the W15 regime was higher than that under the W35 regime for the same biochar amendment, which showed that a greater amount of irrigation and soil moisture were more beneficial to the formation of SOM for these two studied years. Owing to the increase in pH, available N, available P, available K, and SOM by biochar amendment, soil fertility index for each variable generally increased with increasing biochar application rate (**Figure 5**).

### 4.4 Biochar effects on maize water use efficiency under varied irrigation amounts

These studies reported the positive effects of biochar on crop growth and yield under suitable water fertilizer management strategies (Sadaf et al., 2017; Zheng et al., 2017; Ali et al., 2020; Liu et al., 2021). In this study, the LAI of maize plants was improved by biochar treatments for each year; these averaged LAI measurements were higher in the W15 irrigation regime than in the W35 regime for the same biochar treatments (**Figure 5**). Possible reasons for this are high net photosynthesis rate and leaf chlorophyll content under better irrigation conditions with greater available nutrients and organic matter (**Figure 4**). Several studies have shown that LAI is directly related to available N and leaf chlorophyll after the addition of biochar (Agbna et al., 2017; Alkhasha et al., 2019; Yang et al., 2022). However, there were no significant differences in the average LAI values between the B15, B30, and B45 treatment, which revealed that biochar amendment at 15 t ha-1 was suitable for maize growth in terms of aboveground crop growth.

Since greater LAI and better phenotypic performance were measured for biochar amendment, the maize aboveground dry mass were partly increased (**Table 4**). However, there were no significant differences in biomass among the B15, B30, and B45 treatments in either the W15 or W35 regimes, which is in accordance with results obtained by Yang et al. (2020). Under the W15 and W35 regimes, the maize yield in the first year was higher than that in the second year with the same biochar treatment. Main cause for this is that the low rainfall was occurred in late August through early September in the second year (**Figure 1**), which constrained the grain-filling rate and grain weight. Since a greater yield was obtained from the W35 regime compared to the W15 regime for the same biochar amendment (as averaged across two years), the IWP under the W35 regime was greater than that under the W15 regime, while partial fertilizer productivity was not significantly different between the W15 and W35 regimes for the same amendments (**Figure 8**). Therefore, in terms of crop yield and water-fertilizer resource use, we suggest a one-time B15 biochar application in mid-spring with reduced irrigation using a drip fertigation system in the semiarid region of China.

As continuous application higher dose of biochar was considered by most of studies in upland soils, and these have reported some beneficial results for water/fertilizer saving, yield increase, and greenhouse gas reduction with higher input of synthetic fertilizers. It was known that high application rate of biochar could led to more agricultural inputs, so the study mainly focused on a one-time biochar application within two growing seasons for reducing the biochar inputs at the initial of experiment, thus determining the potential and actual effects in soil nutrients improvement, crop growth and yield promotion, water-fertilizer productivity increase under reduced irrigation regime.

Since the complexity of experimental factors on water, fertilizer, and biochar, the study did not consider fertilizer application amount and agricultural inputs. Further we will include fertilizer amount as experimental factor so as to recommend the more reasonable management solution on water, fertilizer, and biochar, proposing the substitution of fertilizer requirement with appropriate biochar doses, thus improving water-fertilizer use efficiency and reducing agricultural inputs under reduced irrigation conditions.

## 5 Conclusion

In the arid and semiarid region, biochar application rates of 15, 30, and 45 t ha^-1^ showed positive potential for improving soil moisture, comprehensive fertility index, above-ground growth and yield, and water-fertilizer productivity for the assigned water lower limits by the drip-fertigation system across over two years. For soil amended with 30 t ha^-1^ and 45 t ha^-1^ in the second year, the soil available N was higher under W15 than under W35, nevertheless the soil available P was higher under W35 than under W15. In the first and second year, the irrigation water productivity respectively improved by 23~29% and 28~31%, in the W15 regime than in the W35 regime when biochar was used. In comparison, a one-time biochar amendment at 15 t ha^-1^ was more suitable and recommended for a film-mulched drip-irrigation (with a water lower limit of -15 kPa soil matric potential and 22.5 mm of water for each irrigation). Since the high dose of biochar was adopted in the cropping system, the agricultural cost and economic benefit should be further studied so as to propose an reasonable solution for biochar and irrigation applications.

## Data availability statement

The raw data supporting the conclusions of this article will be made available by the authors, without undue reservation.

## Author contributions

WY: Writing - original draft, Project administration, Funding acquisition. GF: Writing - review and editing. YJ: Investigation, Methodology, Data curation. XG: Writing - review and editing. LG: Investigation, Methodology, Data curation. ZQ: Project administration, Funding acquisition, Writing - review and editing. All authors contributed to the article and approved the submitted version.

## Funding

This work was supported by National Key Research and Development Program of China (No. 2021YFC3201202), National Natural Science Foundation of China (No. 52109056, 52279037), Inner Mongolia Autonomous Region Research Project “Rejuvenating the Inner mongolia with science and technology”(No. 2021EEDSCXSFQZD011). Natural Science Foundation of Inner Mongolia Autonomous Region of China (No.2021BS05003), and Inner Mongolia Agricultural University High-level Talents Research Project (No. NDYB2020-1).

## Conflict of interest

The authors declare that the research was conducted in the absence of any commercial or financial relationships that could be construed as a potential conflict of interest.

## Publisher’s note

All claims expressed in this article are solely those of the authors and do not necessarily represent those of their affiliated organizations, or those of the publisher, the editors and the reviewers. Any product that may be evaluated in this article, or claim that may be made by its manufacturer, is not guaranteed or endorsed by the publisher.
